# Editorial: Molecular basis of seed germination and dormancy, volume II

**DOI:** 10.3389/fpls.2026.1797892

**Published:** 2026-02-17

**Authors:** Qianya Zhou, Xiaohong Tong, Yong Xiang, Zhoufei Wang, Łukasz Wojtyla, Yifeng Wang

**Affiliations:** 1 State Key Laboratory of Rice Biology and Breeding, China National Rice Research Institute, Hangzhou, China; 2 Shenzhen Branch, Guangdong Laboratory for Lingnan Modern Agriculture, Genome Analysis Laboratory of the Ministry of Agriculture, Agricultural Genomics Institute at Shenzhen, Chinese Academy of Agricultural Sciences, Shenzhen, China; 3 The Laboratory of Seed Science and Technology, Guangdong Key Laboratory of Plant Molecular Breeding, State Key Laboratory for Conservation and Utilization of Subtropical Agro-Bioresources, South China Agricultural University, Guangzhou, China; 4 Department of Plant Physiology, Institute of Experimental Biology, Faculty of Biology, Adam Mickiewicz University, Poznań, Poland

**Keywords:** crop improvement, hormone crosstalk, multi-omics, quantitative trait locus (QTL), seed dormancy, seed germination

Seed germination, dormancy, and vigor are core traits that underpin plant fitness and crop yield. The classical framework of ABA-GA antagonism (Finch-Savage and Leubner-Metzger, 2006) has been enriched by studies showing the integral roles of metabolism, environmental sensing, and hormones such as auxin and brassinosteroids (Nonogaki, 2014; Shu et al., 2016). Modern perspectives emphasize the integration of these regulatory layers, from hormonal signaling to metabolic networks (Carrera-Castaño et al., 2020; Finkelstein et al., 2008). This Research Topic presents six studies that address this challenge by employing cutting-edge techniques to dissect the molecular basis of seed traits from complementary angles.

Integrative multi-omics has emerged as a key strategy for unravelling the complex physiology of seed dormancy. In cereal crops such as wheat and barley, combined transcriptomic and metabolomic analyses have linked dormancy release to specific shifts in hormone and primary metabolism (Gubler et al., 2005; Barrero et al., 2009). The role of seed coat components, particularly flavonoids, in imposing dormancy is a conserved feature highlighted in model systems, and systems biology approaches are powerful for mapping such traits (Righetti et al., 2015). Wang et al. extend this approach to non-model species, the medicinal plant *Smilax glabra*, and provide the first comprehensive, time-resolved multi-omics atlas of dormancy release in this species. By constructing a core “metabolite-gene” coexpression network, it pinpoints the coordinated induction of flavonoid catabolism and GA biosynthesis as a hallmark of the dormancy-to-germination transition, offering concrete metabolic markers and regulatory candidates for propagation control.

Mapping the genetic architecture of seed traits through quantitative trait locus (QTL) analysis is a cornerstone of crop improvement. The successful isolation of QTLs for dormancy and vigor in rice, maize, and Arabidopsis has led to the discovery of major regulators, such as *Delay of Germination 1 (DOG1)* and *Seed Dormancy 4 (SDR4)* (Bentsink and Koornneef, 2008; Sugimoto et al., 2010). Exploring natural variation in model species remains a powerful strategy for dissecting the genetic architecture of complex seed traits. For instance, population-level studies in *Arabidopsis* have revealed fundamental trade-offs, such as a negative correlation between seed longevity and dormancy, highlighting how genetic factors shape overall seed fitness (Nguyen et al., 2012). Building on this paradigm, two studies in this Topic advance precision genetics. Saini et al. leverage a recombinant inbred line population to identify stable QTLs for seed viability in soybean and further prioritize candidate genes functionally enriched for roles in protein repair and oxidative stress response, providing a mechanistic hypothesis for genetic differences in seed aging. Concurrently, another study, Shan et al. employ a set of chromosome segment substitution lines to finely map a major QTL for early germination from the *aus* variety Kasalath in a japonica background, narrowing the interval to genes implicated in starch mobilization and GA sensitivity, thus delivering precise targets for breeding accelerated, uniform emergence.

The hormonal network controlling germination is now recognized as a multi-component web beyond the core ABA-GA axis. Auxin, for example, regulates endosperm cap weakening and embryo growth in tomato and *Arabidopsis* (Liu et al., 2013), with conserved mechanisms, such as phytochrome-interacting factor (PIF)-mediated auxin signaling, which also modulates seed dormancy in cereals like rice (Sun, 2010). The dedicated review by Ament et al. systematically synthesizes evidence across species, positioning auxin not merely as a modulator but as a core component of the germination trigger. It details how auxin signaling intersects with both the GA activation and ABA degradation pathways, thereby refining the classical hormonal framework into a dynamic, interconnected network.

A central goal is to understand how systemic signals are executed at the cellular level to drive radicle emergence. The roles of reactive oxygen species (ROS) and starch metabolism as key executors have been highlighted in *Arabidopsis* and sunflower (Bailly et al., 2008; Leymarie et al., 2012). ROS are dynamically integrated with hormone signals, such as ABA and ethylene, to regulate the germination decision (El-Maarouf-Bouteau et al., 2015), and the transition from dormancy to germination is essentially a ‘wake-up call’ involving these coordinated signals (Née et al., 2017). In this context, the functional study of *OsNUDX23* in rice by Dai et al. employs CRISPR-Cas9 knockout mutants to dissect the gene’s role mechanistically. It demonstrates that *OsNUDX23*, a Nudix hydrolase, is indispensable for maintaining optimal cellular ROS levels and for the rapid degradation of starch during early imbibition. This work elegantly identifies a single genetic node that directly couples oxidative signaling to metabolic energy supply, a critical convergence point for germination.

Beyond ROS and starch, the aleurone layer integrates hormonal signals to regulate germination (Bethke et al., 2007), in part by mobilizing stored lipids to fuel radicle emergence. How this signal transduction links to lipid metabolism remains unclear. To address this, a new study by Cao et al. identifies the aquaporin gene *OsTIP3;1* as a key regulator of lipid metabolism in the rice aleurone layer via transcriptome analysis, which connects the classic role of aquaporins in water transport, essential for imbibition (Maurel et al., 2008) and subject to complex hormonal regulation (Maurel et al., 2021), directly to reserve mobilization. Thus, *OsTIP3;1* emerges as a novel tissue-specific integrator, coordinating water dynamics with lipid metabolism to translate systemic signals into germination action.

In summary, this Research Topic strengthens the molecular foundations of seed biology by bridging scales and species. From the system-level discovery of a flavonoid-GA metabolic switch in *Smilax glabra*, to the identification of candidate genes for soybean longevity and rice early vigor through precise QTL mapping, to the redefinition of auxin’s central role in hormonal crosstalk, and finally to the functional elucidation of key cellular executors in rice, integrating ROS-starch signaling and water-lipid metabolism, these contributions provide specific resources and integrative insights. Cross-species comparisons are vital for distinguishing conserved regulators from lineage-specific adaptations (Graeber et al., 2010). Future research should prioritize comparative validation and the integration of multi-omics datasets with genome editing to enhance seed quality, longevity, and climate resilience. This translational effort finds a clear roadmap in comprehensive reviews that integrate molecular insights with breeding objectives for key crops, such as rice (Xu et al., 2025). We hope this collection stimulates further integrative efforts, accelerating progress toward both fundamental understanding and sustainable agricultural innovation.
